# Sigmoid Volvulus Complicating Postpartum Period

**DOI:** 10.1155/2017/9034925

**Published:** 2017-01-31

**Authors:** Kelsey E. Ward, Erin Blake, Eduardo Gonzalez, Frederic Pieracci, Meredith Alston

**Affiliations:** ^1^Department of Obstetrics and Gynecology, University of Colorado School of Medicine, Aurora, CO, USA; ^2^Department of Surgery, University of Colorado School of Medicine, Aurora, CO, USA; ^3^Department of General Surgery, University of Colorado School of Medicine and Denver Health Medical Center, Denver, CO, USA; ^4^Department of Obstetrics and Gynecology, University of Colorado School of Medicine and Denver Health Medical Center, Denver, CO, USA

## Abstract

*Background*. Sigmoid volvulus is a rare complication of pregnancy and the puerperium.* Case*. A 19-year-old patient, gravida 1 para 0 at 41 0/7 weeks of gestation, admitted for late-term induction of labor underwent an uncomplicated primary low transverse cesarean delivery for arrest of descent. Her postoperative period was complicated by sudden onset of abdominal pain and the ultimate diagnosis of sigmoid volvulus.* Conclusion*. Prompt surgical evaluation of an acute abdomen in the postpartum period is essential; delayed diagnosis and treatment can lead to significant maternal morbidity and mortality.

## 1. Introduction

Sigmoid volvulus is an infrequent complication in the setting of pregnancy. Normal physiological findings of the puerperium such as abdominal pain, nausea, and leukocytosis can obscure the clinical picture in the setting of sigmoid volvulus. However, it is imperative to consider this rare entity in the differential diagnosis of severe abdominal pain as early recognition has proven to be the primary predictor of outcome [1].

## 2. Case

The patient is a 19-year-old gravida 1 para 0, who presented at 41 0/7 weeks of gestation for a late-term induction of labor. The patient complained of lower back and bilateral hip pain associated with contractions upon arrival. Contractions were occurring every 10–15 minutes and had started 3-4 hours prior to her admission. She otherwise denied vaginal bleeding, leaking of fluid, constipation, or diarrhea with normal daily bowel movements.

The patient's medical history and antepartum course were uncomplicated. She had no significant medical or surgical history. She denied past or present substance use. She had immigrated from the Democratic Republic of Congo three years prior to presentation. Her prenatal laboratory values were within normal limits.

Fetal status on arrival was reassuring with a category 1 fetal heart tracing. Ultrasound confirmed adequate fluid and cephalic presentation. Induction was initiated with oral misoprostil followed by spontaneous rupture of membranes notable for meconium staining. Augmentation then proceeded with intravenous oxytocin. The sterile vaginal exam was notable for the fetal head in the right occiput posterior position and an estimated fetal weight of 3700 grams. An attempt at manual rotation was unsuccessful. After 24 hours of induction and 12 hours following rupture of membranes, maternal fever rose to 38.5 with fetal tachycardia and the patient was started on ampillicin-sulbactam for a diagnosis of chorioamnionitis. The patient reached full dilation. After four hours of pushing, the decision was made to proceed with primary low transverse cesarean delivery for arrest of descent. A vigorous male infant with Apgar's of 7 and 9 at 1 and 5 minutes was delivered without difficulty; however the cesarean section was subsequently complicated by significant uterine atony and the patient received 250 micrograms (mcg) of intramuscular carboprost, 800 mcg rectal misoprostil, 0.2 milligrams (mg) intramuscular methylgonervine and 80 units intravenous oxytocin. The atonic uterus was reduced with a B-Lynch suture using 0 chromic suture and the estimated blood loss was 1500 milliliters.

Approximately 15 hours following surgical closure, the patient developed acute abdominal pain with marked distention, rebound, and guarding. Bedside abdominal ultrasound was performed secondary to concern for intra-abdominal bleeding with no evidence of intraabdominal fluid, but the ultrasound evaluation was significantly limited due to patient discomfort. Vitals were notable for tachycardia to 109 and fever to 38.4°C with no evidence of hypotension or hypoxia. The patient was taken for emergent exploratory laparotomy due to her acute abdominal examination with no preoperative imaging done due to acuity.

Upon opening, a moderate amount of intra-abdominal serosanguinous fluid was noted without significant hemoperitoneum. The uterine incision was hemostatic and no obvious uterine abnormalities were noted. Upon inspection of the bowel, the sigmoid colon was noted to be dilated to greater than 10 centimeters in diameter. General surgery was consulted intraoperatively and a volvulus at the mesenteric root of the dilated sigmoid colon was diagnosed. The sigmoid was subsequently reduced by performing a 180-degree manual rotation. The entirety of the bowel was inspected and noted to be mildly edematous and dilated; however, brisk mesenteric pulses were noted with no areas of necrosis or perforation. At this point, a 36 Fr straight lubricated chest tube was inserted rectally and passed without resistance to the level of the sigmoid colon under direct palpation. Suction was applied in order to decompress the colon. The bowel was again inspected and volvulus remained resolved. The Pfannensteil incision was closed in usual fashion.

Following 24 hours of decompression, the rectal tube was removed. The patient was diagnosed with symptomatic anemia of acute blood loss based on a hematocrit of 21.8, fatigue, and dizziness with ambulation. She received two units of packed red blood cells with appropriate improvement in hematocrit and symptoms. The patient was also febrile to 38.2°C on postoperative day #2 and was treated with 24 hours of intravenous clindamycin and gentamicin with resolution of fevers. The patient was discharged on postoperative day four meeting all postoperative milestones including passing flatus and tolerating a regular diet. Her subsequent postoperative course was uncomplicated.

## 3. Comment

This case represents an unusual case of postpartum sigmoid volvulus. The most common causes of intestinal obstruction in the puerperium are adhesions (58%), volvulus (24%), intussusception (5%), hernia, carcinoma, and appendicitis [2, 3] with sigmoid volvulus being the most common volvulus found surrounding pregnancy. This phenomenon was first reported by Houston in 1830 and initial maternal and fetal mortality was described in 1937 with rates of 21% and 50%, respectively [1]. In other reports, maternal and fetal outcomes were directly related to bowel ischemia and all maternal deaths were associated with delayed presentation and surgical intervention over 48 hours [1, 4, 5]. Maternal mortality has improved since initial description of volvulus in pregnancy from 1830; however mortality rates are still unacceptably high with a 20% maternal and 40% fetal mortality [1, 6]. A review of intestinal obstruction complicating the puerperium found overall maternal and fetal mortality from volvulus to be 13% and 20%, respectively, which is a higher mortality rate than any other cause of intestinal obstruction [1].

Volvulus in the postpartum period, as in this case, is exceedingly rare, but risk is thought to be increased from distortion of the colon occurring from rapid change of uterine size following delivery [7]. This risk may be further increased by an underlying redundancy of the sigmoid colon in the setting of a narrow-based mesentery. The triad of symptoms associated with volvulus is traditionally pain, distention, and obstipation. Nausea, vomiting, hyperkinetic, or hypokinetic bowel sounds are common secondary symptoms and signs [1, 5, 8]. However, these symptoms are nonspecific and are common in the postpartum period. The uterus, cervix, and adnexa share the same visceral innervations as the terminal ileum, sigmoid, colon, and rectum so differentiation between a gynecologic or gastrointestinal origin for pain can be difficult [8]. Furthermore, abdominal distention may be a late and unreliable finding of postpartum volvulus and associated abdominal tenderness and, if found, may be interpreted as fundal tenderness since the obstructed intestine is typically posterior to the enlarged uterus [9]. Postpartum, the clinical picture may be confused with endometritis or postoperative complications.

The diagnosis of volvulus, including in the postpartum period, requires a high index of suspicion and it has been shown that prompt intervention is the best predictor of improved patient outcomes. In the 84 cases reviewed, the delay in onset of symptoms to diagnosis ranged between a few hours to as many as 6 days [10]. Ancillary testing that may be of value includes abdominal XRAY, barium enema, and colonoscopy. However, in the setting of an acute abdomen, immediate surgical exploration is warranted. In 300 cases of colonic volvulus reviewed by Mayo clinic, mortality was 80% in patients with gangrenous bowel compared to 10.6% in patients with viable colon [11].

Nonoperative reduction of the sigmoid volvulus delivers adequate decompression of the bowel but does not prevent future episodes; in fact, 90% of patients treated with conservative reduction have been shown to have recurrence [11]. The operative management of acute sigmoid volvulus depends upon the viability of the bowel, physiologic status of the patient, and likelihood of recurrence. Cases of primary sigmoid volvulus are typically seen in elderly patients with chronic constipation. In this case, the likelihood of recurrence is high, and sigmoid resection is recommended. Although cases of secondary volvulus, as was seen in this patient, are rare, it is believed that the risk of recurrent volvulus is far lower, as resolution of the inciting etiology (in this case, changes in intraabdominal pressure and organ location) will likely minimize the risk of recurrent volvulus. However, the long term risk of recurrence in patients with pregnancy-associated sigmoid volvulus remains unknown. As the incidence of major morbidity (including necessity for colostomy) following urgent or emergent sigmoid resection approaches 40% [12], we recommend decompression alone for cases of pregnancy-associated sigmoid volvulus. Patients with primary sigmoid volvulus have a higher risk of recurrence and definitive surgical management to prevent recurrence may be recommended following decompression [11]. However, given that secondary recurrence of sigmoid volvulus is thought to be much lower, this patient will follow up with general surgery for postoperative care with no further surgical intervention unless recurrence or further complications are suspected.

In patients presenting with acute abdomen in the postpartum period, as our patient, prompt evaluation and radiologic imaging may allow for timely diagnosis. In this case, no imaging was ordered since the clinical recognition of an acute abdomen requiring surgical management called for emergent intervention. The time from symptom onset to intervention was less than two hours which was likely the main contributor to the preservation of bowel without need for resection.

## Additional Points

*Teaching Points*. (1) Worsening abdominal pain in the postpartum and postoperative state should prompt rapid evaluation. (2) Timely intervention for sigmoid volvulus is warranted since delay in recognition and treatment has proven to rapidly increase morbidity and mortality.
